# Fault Detection of Roller-Bearings Using Signal Processing and Optimization Algorithms

**DOI:** 10.3390/s140100283

**Published:** 2013-12-24

**Authors:** Dae-Ho Kwak, Dong-Han Lee, Jong-Hyo Ahn, Bong-Hwan Koh

**Affiliations:** Department of Mechanical, Robotics and Energy Engineering, Dongguk University-Seoul, 30 Pildong-ro, 1-gil, Jung-gu, Seoul 100-715, Korea; E-Mails: daeho860@hanmail.net (D.-H.K.); micro89@hanmail.net (D.-H.L.); dkswhd83@gmail.com (J.-H.A.)

**Keywords:** roller-bearing, fault detection, minimum entropy deconvolution, genetic algorithm

## Abstract

This study presents a fault detection of roller bearings through signal processing and optimization techniques. After the occurrence of scratch-type defects on the inner race of bearings, variations of kurtosis values are investigated in terms of two different data processing techniques: minimum entropy deconvolution (MED), and the Teager-Kaiser Energy Operator (TKEO). MED and the TKEO are employed to qualitatively enhance the discrimination of defect-induced repeating peaks on bearing vibration data with measurement noise. Given the perspective of the execution sequence of MED and the TKEO, the study found that the kurtosis sensitivity towards a defect on bearings could be highly improved. Also, the vibration signal from both healthy and damaged bearings is decomposed into multiple intrinsic mode functions (IMFs), through empirical mode decomposition (EMD). The weight vectors of IMFs become design variables for a genetic algorithm (GA). The weights of each IMF can be optimized through the genetic algorithm, to enhance the sensitivity of kurtosis on damaged bearing signals. Experimental results show that the EMD-GA approach successfully improved the resolution of detectability between a roller bearing with defect, and an intact system.

## 1. Introduction

As modern industries inevitably utilize a wide range of rotating machinery, the imperative of securing its safety during the service life has also escalated significantly. In particular, the maintenance and repair costs due to degradation of a system have increased, along with the functional complexity of the mechanical systems. In fact, sudden failure or structural defects may lead to catastrophic accidents. Thus, the potential threat to the economy and human losses has increased. In the case of rotating machinery, real-time vibration analysis and feature extraction techniques are required to identify the incipient defects, caused by fatigue, contamination, overload or poor maintenance. In this context, vibration-based preemptive condition monitoring schemes for rotating machinery have been actively studied since the 1970s [1–3].

One of the most widely used vibration-based techniques is to compare the trend of shifted harmonics or kurtosis, which evaluate the frequency and time-series measurements, to determine the presence of defects [4,5]. The kurtosis of a given signal, defined as the fourth standardized moment of a distribution, statistically expresses the level of sharpness in its peaks of waveform. For a normal distribution, the value of kurtosis becomes three. Commonly, mechanical defects, such as cracks and/or spalling on the surface of the rolling element, create repetitive impulses, when they collide with the adjacent rotating components. Obviously, the impact wave from this collision passes through the whole system, and its resonated pulses change the kurtosis value. However, if the extent of the defect is relatively small, significant changes in the value of kurtosis may not be observed [6]. Hence, it is necessary to apply appropriate signal processing methods, to enhance the sensitivity of kurtosis to the presence of defects in rotating machinery.

Among many signal processing techniques, deconvolution-type methods have attracted extensive attention in recent years. The minimum entropy deconvolution (MED) technique was first proposed by Wiggins, for extracting reflective information from seismic recordings [7]. The deconvolution process separates the seismic reflection, by minimizing the entropy of the signals. The MED inherently exploits a norm that represents the simplicity of the vector. Here, the simplicity represents orthogonal vectors, whose normalized variance should be maximized, which is also known as varimax rotations [8–11]. In the process of finding maximum variance, optimized MED filter coefficients can be iteratively found. Although the original use of MED was for signal processing of seismic data, this deconvolution technique has been extended to other applications: multisensor data fusion [12], pitch period estimation [13], and the restoration of star field images [14]. In regards to machinery diagnosis, Endo and Randall explored the application of MED to enhancing the effectiveness of filters in the fault detection problem [15]. Notably, Sawalhi *et al.* [16] presented a study on bearing fault detection using the spectral kurtosis in conjunction with linear prediction filtering and MED.

Moreover, one of the modulation techniques, also known for Teager-Kaiser Energy Operator (TKEO) has been applied for monitoring abrupt change of energy in neurological signals [17]. The concept of TKEO was first proposed by Teager [18] in 1980, for modeling nonlinear speech production. Later, Kaiser [19] applied the TKEO to single time varying signals, for simultaneous modulation of amplitude and frequency. This signal conditioning method has shown successful results in fault detection of a rotating machinery [20,21].

Huang [22] proposed empirical mode decomposition (EMD) as an essential component of time-frequency analysis. EMD is an excellent signal processing tool that decomposes an arbitrarily signal into finite number of modes or intrinsic mode functions (IMF) regardless of linear or stationary nature of the signal [23]. Many studies have exploited the IMF of vibration signals from rotating machinery for condition monitoring and fault detection [24]. However, most of the approach has been focused on analyzing the characteristics of individual IMF to extract fault-sensitive features. Celebi [25] has shown that the quality of underwater images could be enhanced through EMD and genetic algorithm (GA). He first decomposed the image or 2D matrix into multiple IMFs (intrinsic mode functions), using EMD. Then, coefficients or weights were imposed on each IMF of pixel vectors. Finally, the GA optimized the weight of IMFs to improve the image quality, through minimizing the entropy of the pixel. This concept can be applied to the bearing fault detection by finding an optimal set of weights to maximize the differences of kurtosis value between the healthy and damaged bearing condition.

In this study, we investigate two different signal processing methods of vibration signals of bearing, with attempts to identify the characteristics of defects: MED [7], and the TKEO. Also, the study compares the kurtosis value of the measured vibration signals using a fused optimization tool, *i.e.*, a GA combined with EMD [25], to enhance the sensitivity of defects in a roller-bearing. This paper is organized as follows: first, vibration signals from laboratory-based bearing-rotation tests are collected, to compare the kurtosis value before and after the infliction of scratch-type defects on the surface of the inner race of bearings. Secondly, the performances of two data processing techniques, *i.e.*, MED and the TKEO are discussed, in terms of the sequence of applications towards vibration data. Finally, EMD is combined with GA, to optimize the weight of each IMF, to maximize the resulting kurtosis values.

## 2. Background Theories

### 2.1. MED (Minimum Entropy Deconvolution)

In the 1980s, Wiggins first introduced the concept of Minimum Entropy Deconvolution (MED) that maximizes spike-like characteristics from noise-corrupted data points, or equivalently minimizes the entropy of signal components, using a linear operator [7]. The theoretical background of MED begins with the deconvolving process of seismic reflectivity from measurements. Given *g* as an arbitrary transfer function representing all the paths between the rolling element of bearing and the sensor location, the measurement *w_i_* with additive noise *n_i_*, can be expressed as a convolution of Equation (1):

(1) $$w_{i}=g\times q_{i}+n_{i}$$

here, *q_i_* denotes the source signal generated by the collision between rolling element and the defect on the bearing surface. Hence, the goal is simply recovering or deconvolving the source signal *q_i_* from noise-corrupted measurements, without the *a priori* knowledge of *g*. Although the underlying theory of solving this problem through MED is similar to that of blind source separation [26,27], the constraint towards the coefficient of the deconvolution filter is different, as the algorithm finds a solution to minimize the entropy of the signal. In information theory, the entropy represents the amount of data content in the sequence of a given signal [12]. Thus, a higher level of entropy is associated with the enforcement of randomness or uncertainty. In contrast, minimizing the entropy leads to a “*simple*” structured signal, such as sparse spikes. Again, it is critical to recover the impact or spike-like segments from the sensor signal, for successful condition monitoring of a roller-bearing. If we consider an input *q_i_* (*q_1_*, …, *q_N_*), and pass them to filter *f*, then:

(2) $$\begin{array}{cc} y=f\times w_{i}=(f\times g)\times q_{i}+\text{f}\times n_{i} & \left(i=1,\ldots N\right) \end{array}$$

Notably, the coefficient of the filter *f* should be chosen such that it satisfies $f\times g=q_{i}^{-1}$ and hopefully minimizes *f* × *n_i_*. This can be indirectly achieved, by defining the varimax criterion as in Equation (3) below, and maximizing its norm:

(3) $$V(y)=\sum_{1}^{N}{\left(\frac{y_{i}}{\left\|y\right\|}\right)}^{4}$$

To achieve this, the varimax norm needs to be differentiated with respect to filter coefficients, or $\frac{\partial V}{\partial f_{k}}=0$. Finally, equations can be developed in matrix form:

(4) R(f)×f=ξ(f)

where, *R*(*f*) and *ξ*(*f*), indicate an autocorrelation matrix of input sequences and filter-dependent cross-correlation vector, respectively. Because of the nonlinear nature of Equation (4), an iterative algorithm is required to yield the solution.

**Figure 1.**
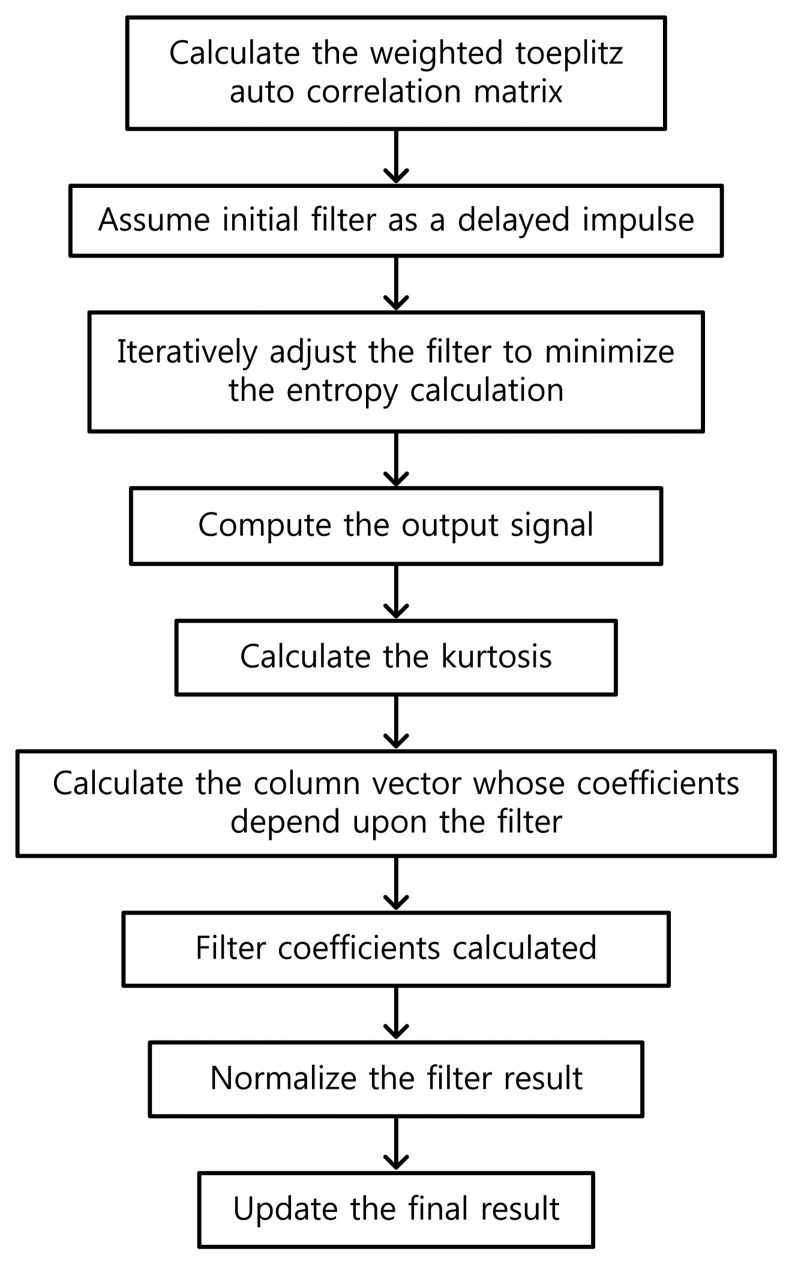
A systematic block diagram to illustrate the process of minimum entropy deconvolution (MED).

As depicted above, the MED algorithm minimizes the entropy of an arbitrary input signal through the deconvolution process, so that the kurtosis of the signal can be maximized. Figure 1 illustrates the conceptual flowchart of MED. In this study, MED will be utilized to detect the bearing faults, in conjunction with kurtosis optimization.

### 2.2. TKEO (Teager-Kaiser Energy Operator)

The TKEO algorithm belongs to the category of nonlinear high-pass filters, which reduce the variation of low frequency background signals, while boosting transient components of a signal in the high frequency region. Eventually, transients and background signals can be easily separated through the TKEO. The first-order discrete time model of the TKEO is expressed as Equation (5):

(5) $$\psi (x[n])=x^{2}[n]-x[n-1]x[n+1]$$

here, n denotes the current time step, and *x*[*n*] indicates the discrete signal. Thus, *ˆ*(*x*[*n*]) represents the n-*th* sampled signal that passed the TKEO, also known as the Teager Energy of the signal. Because the TKEO detects a sudden change of the energy stream without any assumption of the data structure, this operator can be used for condition monitoring of a non-stationary signal. In this study, the TKEO will be applied to amplify the transient or impulse component caused by defects on bearings, and suppress the background noise, to increase the kurtosis sensitivity.

### 2.3. EMD (Empirical Mode Decomposition)

Given an oscillatory data set, EMD iteratively separates high frequency components from the original data, by enforcing the condition of IMF. Specifically, the core of EMD is the sifting process, which first defines envelopes of upper and lower extremes, and iteratively subtracts the mean of both envelopes from the previous data set or residual, until it satisfies the necessary condition of IMF. The first condition of IMF begins with the fact that any oscillatory signal, regardless of stationarity or non-stationarity, can be decomposed into separate constituent functions that have a number of extrema (both maxima and minima) and zero-crossings that must be equal, or differ at most by one. The second condition is that the mean value of the envelope defined by the local maxima and the local minima should be zero at an arbitrary point on the IMF.

The process of EMD [22] is as follows: (i) given a data set, find all the local maxima, and connect those points to develop an upper envelope, using a cubic spline. Likewise, find the lower envelope from local minima. (ii) If the mean value of the upper and lower envelopes is denoted as *m_1_*, the difference between the original signal *x*(*t*) and *m_1_* becomes the first component *h_1_*, as below:

(6) $$x(t)-m_{1}=h_{1}$$

If *h_1_* does satisfy the above two IMF conditions, *h_1_* will be the first IMF. Otherwise, new maxima and minima shall again be identified, and steps (i) and (ii) will be repeated, as below. This repeated process is known as sifting:

(7) $$h_{1}-m_{11}=h_{11}$$

If the sifting process is successfully completed after the k-*th* iteration, we will get the first IMF *h_1k_*, as shown in Equation (8):

(8) $$h_{1(k-1)}-m_{1k}=h_{1k}$$

Or equivalently:

(9) $$c_{1}=h_{1k}$$

Again, as *c_1_* satisfies the conditions of IMF, we can separate *c*_1_ from the original *x*(*t*), to extract the second IMF:

(10) $$r_{1}=x(t)-c_{1}$$

here, we assume *r_1_* to be the original signal, and repeat the previous steps to find the second IMF *c_2_*. After the n-*th* iteration, the original signal *x*(*t*) can be decomposed into n number of IMFs, as below:

(11) $$\{\begin{array}{c} r_{1}-c_{2}=r_{2}\\ \vdots \\ r_{n-1}-c_{n}=r_{n} \end{array}$$

Eventually, the decomposition process ends when *r*_n_ becomes a monotonic function (contains no more than two extrema), so that no more IMF can be deduced. Equation (12) expresses the overall process of EMD:

(12) $$x(t)=\sum_{j=1}^{n}c_{j}+r_{n}$$

**Figure 2.**
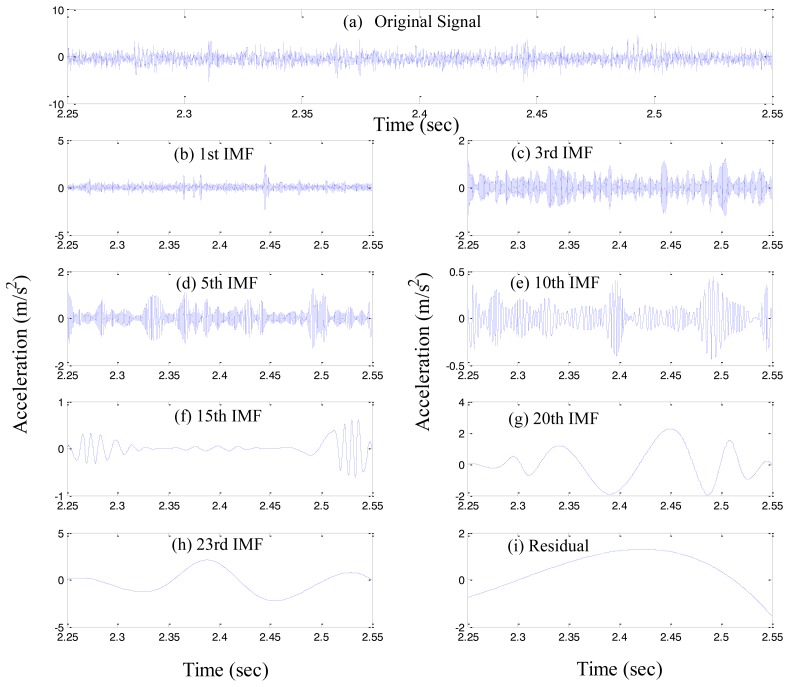
Acceleration measurements of a roller bearing with (**a**) inner-race defect, and (**b**)–(**i**) representative IMFs, after decomposition through EMD.

Thus, we found *n* number of empirical modes, and the residual component *r*_n_. Here, *r*_n_ represents the mean trend of *x*(*t*). The individual IMF, *i.e.*, *c_1_, c_2_*, …, *c_n_* covers a broad range of the frequency band, from the highest to the lowest. Figure 2 illustrates an example of the EMD process, where Figure 2a shows the original signal, and Figures 2b–i depict the decomposed seven IMFs, and the residual.

## 3. Experimental Verifications

### 3.1. A Roller-Bearing and Apparatus

**Figure 3.**
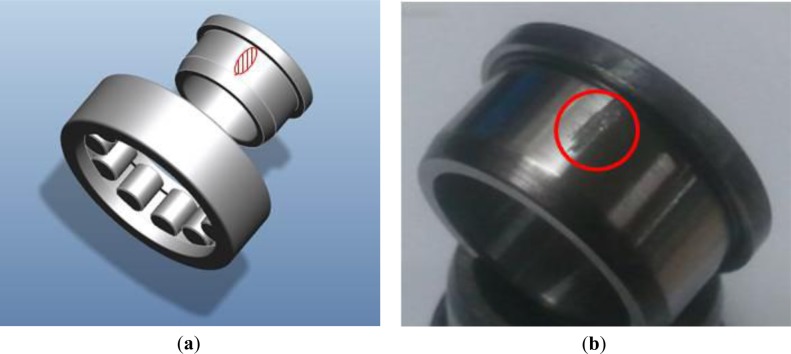
(**a**) A schematic of roller-bearing, and (**b**) a surface scratch on the inner race.

**Figure 4.**
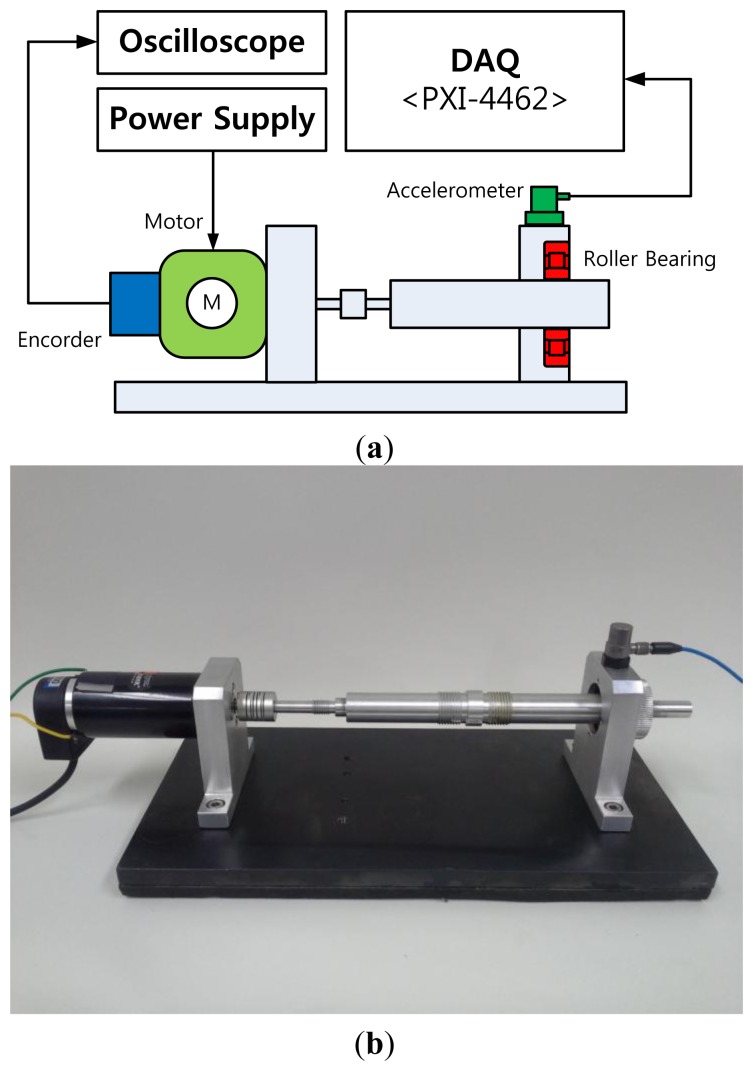
(**a**) Schematic configuration of a bearing test bench and (**b**) the experimental setup.

This section describes an experimental setup and test procedure for a bearing monitoring system. A NJ202ECP roller bearing from SKF was used for the test, as shown in Figure 3. This bearing sustains 12.5 kN of loading, and a shaft speed of 22,000 rpm. In this experiment, a scratch-type defect is caused on the surface of the inner-race of the bearing, as shown in Figure 3b. Figure 4 describes the test setup for measuring vibration data, using a PCB accelerometer (500 mV/g) mounted in the vertical direction of the bearing housing. The vibration signals are recorded with a sampling frequency of 50 kHz, through a LabVIEW-based interface on an NI PXI-4462 system. The speed of the shaft rotation is controlled using an optical encoder.

### 3.2. MED and TKEO-Based Signal Processing

**Figure 5.**
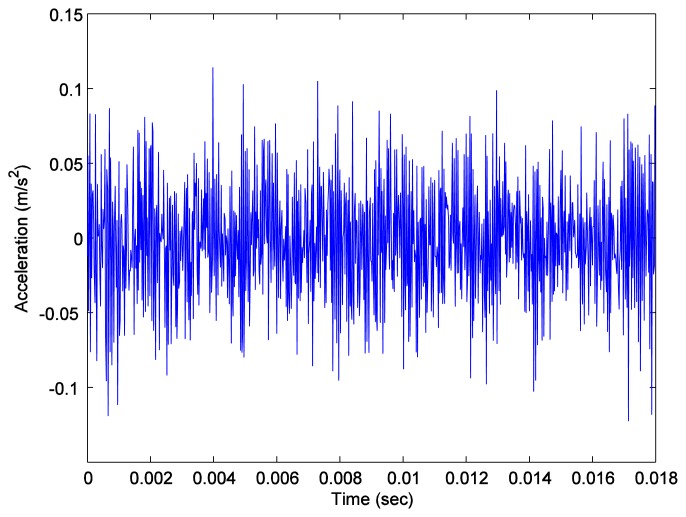
Vibration data points of a roller-bearing with defect (D1).

**Figure 6.**
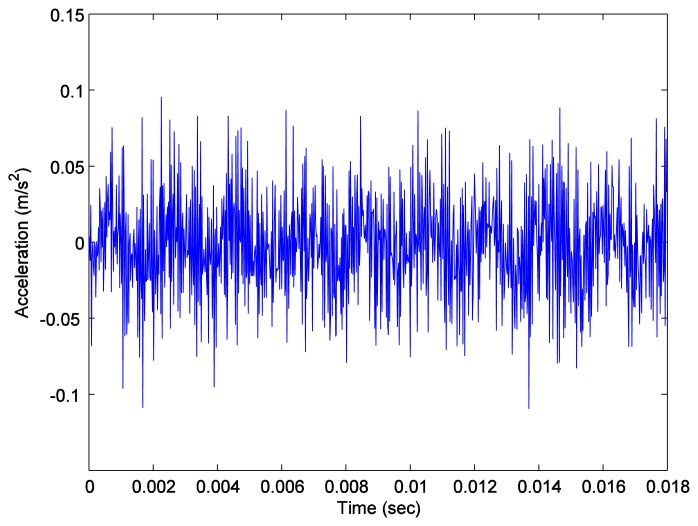
Vibration data points of a roller-bearing in healthy condition (H1).

Again, we measured the vibration signals from accelerometers mounted on the top of the bearing housing. Signals from the bearing with and without defect are compared, for verifying the fault detection methods. Figures 5 and 6 depict segments of acceleration measurements from healthy (H1) and damaged (D1) bearings, respectively. The segment is composed of vibration signals having 2^14^ data points (50 kHz sampling frequency), and we applied algorithms of MED and the TKEO on the segment. In total, five cases are considered; Case I for the original signal, Case II for only MED, and Case III for only the TKEO. We also connected two algorithms in series for Case IV, and switched the sequence for Case V (see Table 1). After that, the kurtosis values of the segment from two healthy and four damaged bearings are calculated. Here, we would be able to compare the performance of each algorithm for monitoring the defect in bearings. Figure 11 illustrates the kurtosis values of healthy (H1, H2) and damaged (D1–D4) bearings, for all five cases (Cases I–V).

**Figure 7.**
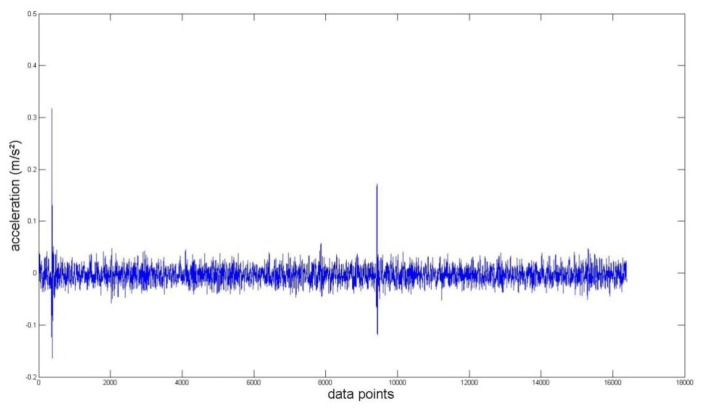
Vibration data points after applying only MED signal processing on a damaged bearing (Case II).

**Figure 8.**
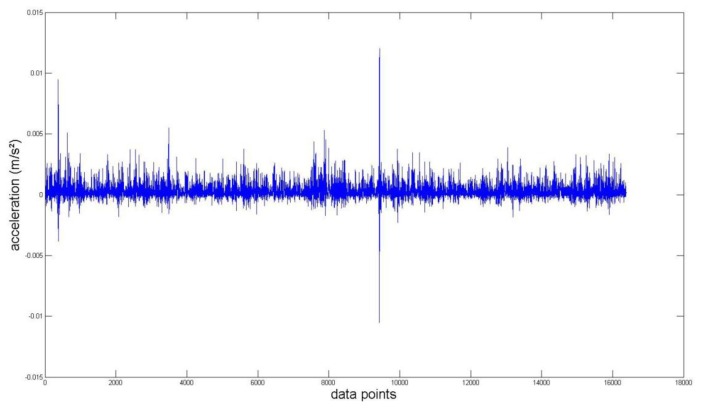
Vibration data points after applying only TKEO signal processing on a damaged bearing (Case III).

**Figure 10.**
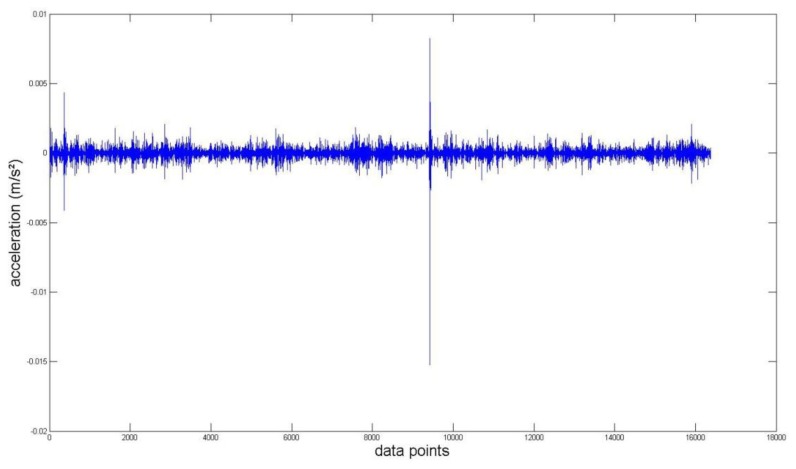
Vibration data points after applying the TKEO first, and then MED signal processing on a damaged bearing (Case V).

**Figure 11.**
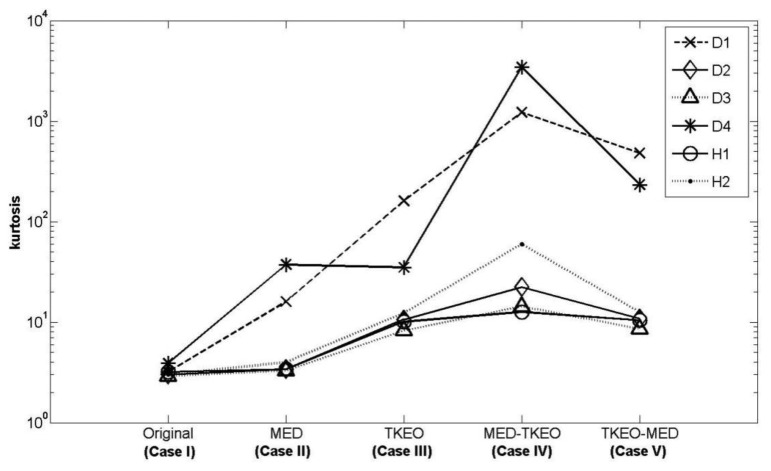
Comparison of kurtosis values after applying different signal processing cases (I–V) on two healthy (H1, H2), and four damaged (D1–D4) bearing data sets.

Having finished MED/TKEO-based signal processing, the results of Cases II–V are shown in Figures 7^8^, 9 and 10, respectively. Again, in Case IV, where vibration data first passed through MED (see Figure 8), quite a different pattern is exhibited from Case V of performing the TKEO algorithm first, as shown in Figure 10. Figure 11 compares all five cases toward different bearing conditions, in terms of kurtosis variation. Surprisingly, one of the damaged bearings D1 shows an enhancement of kurtosis of up to 50-fold after the TKEO. Moreover, if we preprocess MED before the TKEO, its kurtosis is boosted nearly several hundred times higher than the original segment. On the other hand, the healthy bearing condition of H1 exhibits only three times and four times increase in kurtosis, after TKEO and MED-TKEO processing, respectively.

It is apparent that applying MED and the TKEO will significantly increase the kurtosis for the segments from damaged bearings, compared to healthy ones. Interestingly, the MED-TKEO provides a better separation capability than the TKEO-MED process. However, it needs to be also noted that the MED and TKEO processes are not very effective for D2 and D3, as shown in Figure 11. For example, H2 yields slightly higher kurtosis than D2 and D3 for Case IV, which undermines the reliability of MED-TKEO. It is considered that a scratch-type defect on the surface of the outer race produces a unique signature on the vibration measurement for different bearings. This randomness of defect may affect the performance of the aforementioned fault detection algorithms.

### 3.3. Fault Detection through EMD-GA

In the previous section, we compared and discussed the effectiveness of MED and TKEO for diagnosing scratch-type defects on inner-race bearings. Here, we introduce a bearing diagnosis algorithm that combines empirical mode decomposition (EMD) with a genetic algorithm (GA), to maximize the damage-sensitive feature or kurtosis level of given bearing signal. In this study, we used EMD-GA to improve the detectability of bearing faults.

**Figure 12.**
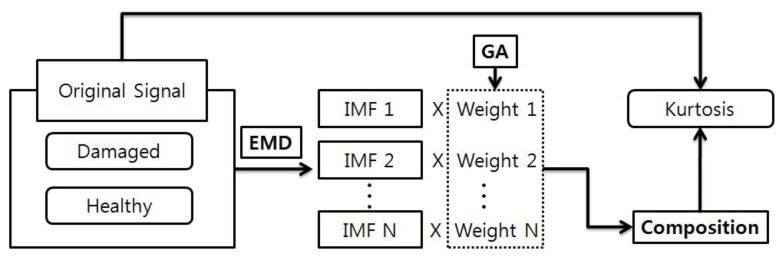
A schematic block diagram for the process of sensitivity enhancement of a bearing defect through EMD-GA.

Figure 12 explains the schematics of EMD-GA that amplifies the kurtosis level, by optimally adjusting the weight of individual IMFs. In order to verify the performance of the EMD-GA approach, segments of signals from both healthy and damaged bearings are decomposed into IMFs. The GA seeks for the optimal weights of the IMF. Here, the objective function to be minimized becomes the negative kurtosis value of the reconstructed segment, when all the IMFs with weights are summed. Apparently, the design variables are elements of the weight vector for each IMF. Having converged IMF weights, we compared the kurtosis of the reconstructed segment from the healthy and damaged bearings. Thus, it becomes possible to increase the contrast of kurtosis between the healthy and damaged bearing signals, after performing EMD-GA.

**Figure 13.**
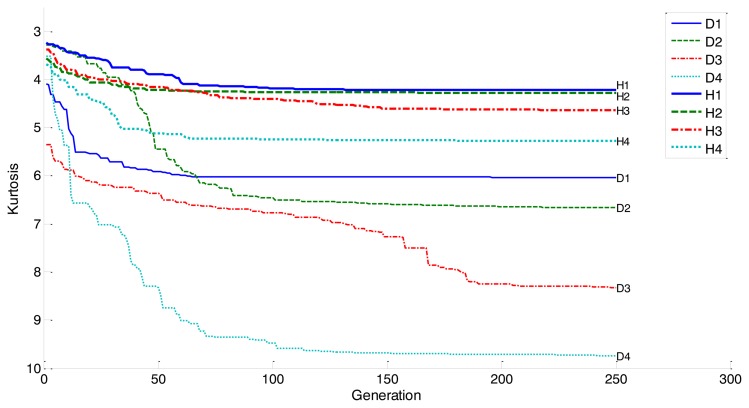
Comparison of the convergence rates of EMD-GA on a single healthy (H1–H4), and four damaged bearing cases (D1–D4).

First, we calculated the IMFs of eight segments of signal, *i.e.*, four healthy (H1–H4), and four damaged bearings (D1–D4). The GA seeks for the vectors of weight for each IMF that maximizes the kurtosis. Having completed the GA, we found that the contrast of kurtosis between healthy and damaged bearings became significant. For this, we set the optimization parameters as follows: population size of 150, mutation rate of 0.2, and maximum iterations are limited to 250. Figure 13 depicts the results of kurtosis variation, as the generation increases. Initially, H1–H4 and D1–D4 have a similar level of kurtosis, as shown in the figure. However, the kurtosis of the initial D4 has dropped by three times, after 250 iterations. On the other hand, the kurtosis of H1 has changed only 40%. Obviously, the contrast has increased, as the EMD-GA is applied to the segments between healthy and damaged bearings.

**Figure 14.**
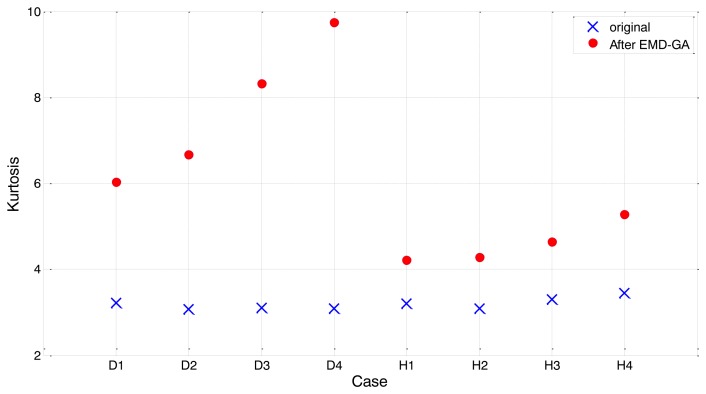
Overall comparison of kurtosis values before and after applying EMD-GA on the bearing signals.

Figure 14 shows the comparison of kurtosis between the original segment and the EMD-GA employed one. As shown in the figure, kurtosis values of the bearing signal segments are not much different in the beginning, for both healthy and damaged bearings. After EMD-GA operation to optimize the weights of individual IMFs, the kurtosis for damaged segment (D1–D4) increases from twice to three times higher than the original. Such an enhancement is quite noticeable, compared to kurtosis variation of a healthy segment (H1), which is around 40%. It should also be noted that the kurtosis values of damaged segments exhibit a relatively large scattering, compared to the original ones. For example, the effect of contrast enhancement for D1 appears much smaller than other segments, such as D3 and D4. Because the severity of defect is unique for each bearing unit, the performance of EMD-GA could be inconsistent for other damaged segments. Again, the gap of kurtosis value between the healthy and damaged bearings is not obvious, as we have seen from Figure 14. This indicates that the kurtosis *per se* does not effectively identify the presence of defect in a bearing. Thus, the performance of condition monitoring towards the early stage of bearing defects can be improved by exploiting the EMD-GA.

## 4. Conclusions

This study presents a comparative study on the condition monitoring of roller bearings through signal processing and optimization techniques. Although it is widely known that the kurtosis values of a bearing with a defect are higher than those of signals from healthy bearings, in many cases the difference is not very obvious. This study suggests and compares two different signal processing techniques (MED and the TKEO), and their combinations, to enhance the resolution of kurtosis, for differentiating the condition of roller-bearing in terms of kurtosis. Experimental results indicate that combining MED and the TKEO successfully improves the resolution of kurtosis for scratch-type defects on the surface of the inner-race of bearings. Also, each segment of the bearing vibration signals can be decomposed into a linear combination of IMFs, using EMD. We employed a GA to optimize the weights of IMFs, to reconstruct the modified segment, providing improved kurtosis sensitivity toward bearing signals with defects on the inner-race. The laboratory study found that the EMD-GA effectively increased the kurtosis sensitivity up to six times on a damaged bearing, while only 40% growth was witnessed on the segment from a healthy bearing. Apparently, the performance of kurtosis-based fault detection for roller bearings can be significantly enhanced by proper selection of data processing and optimization techniques.

## Figures and Tables

**Figure 9.**
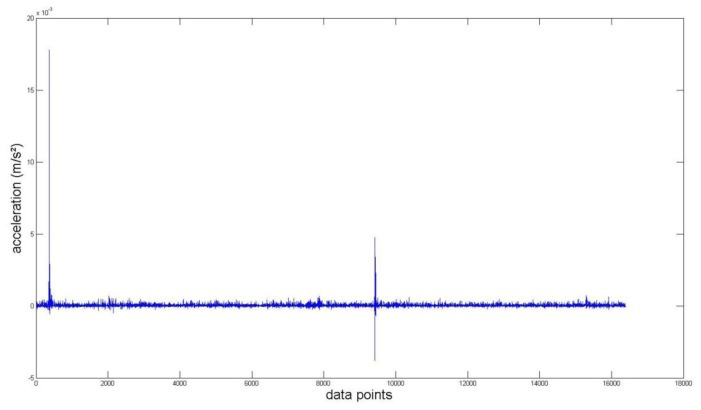
Vibration data points after applying MED first, and then TKEO signal processing on a damaged bearing (Case IV).

**Table 1.** Five different Cases (I–V) of signal processing before calculating kurtosis.

Case	Signal Processing
I	Bearing signal → Kurtosis
II	Bearing signal → MED → Kurtosis
III	Bearing signal → TKEO → Kurtosis
IV	Bearing signal → MED → TKEO → Kurtosis
V	Bearing signal → TKEO → MED → Kurtosis
